# Different situations of identifying second primary malignant tumors in lymphoma patients with synchronous solid tumors

**DOI:** 10.1002/cam4.5592

**Published:** 2023-01-09

**Authors:** Hongye Gao, Xiaogan Wang, Yumei Lai, Chen Zhang, Lan Mi, Xinqiang Ji, Xiaopei Wang, Yuqin Song, Jun Zhu, Weiping Liu

**Affiliations:** ^1^ Key Laboratory of Carcinogenesis and Translational Research (Ministry of Education), Department of Lymphoma Peking University Cancer Hospital & Institute Beijing China; ^2^ Key Laboratory of Carcinogenesis and Translational Research (Ministry of Education), Department of Pathology Peking University Cancer Hospital & Institute Beijing China; ^3^ Key Laboratory of Carcinogenesis and Translational Research (Ministry of Education), Department of Medical Record Statistics Peking University Cancer Hospital & Institute Beijing China

**Keywords:** cancer treatment, lymphoma, survival, synchronous multiple primary neoplasms

## Abstract

**Background:**

To our knowledge, the different situations of identifying second primary malignant tumors (SPMTs) in lymphoma patients with synchronous solid tumors remain to be comprehensively investigated.

**Methods:**

We retrospectively collected information pertaining to lymphoma patients with synchronous solid tumors (diagnosed within 6 months) at Peking University Cancer Hospital & Institute between 2009 and 2019. The non‐parametric Aalen–Johansen estimator was applied to calculate cumulative incidence function in the competing risk model. Furthermore, propensity score‐matched analysis was performed to compare survival differences in lymphoma patients with or without synchronous solid tumors.

**Results:**

Thirty‐eight patients were enrolled. There were three situations of identifying SPMTs. First, in 15 patients (39.5%), SPMTs were identified before the initiation of any treatment. Among them, priority was given to anti‐lymphoma treatment in case of only three patients. Second, in 17 patients (44.7%), SPMTs were unexpectedly detected on surgical specimen assessment; of them, 13 received anti‐lymphoma treatment after surgery. Third, in six patients (15.8%), SPMTs were identified after the outset of treatment for the primary tumor; in this population, three of four patients with lymphoma switched toward the treatment plan for SPMTs. The 5‐year overall survival was 58.7%. The cumulative incidence function within 5 years was 26.6% for lymphoma and 14.7% for other solid tumors. The early identification of SPMTs was associated with better outcomes (*p* = 0.048). After balancing the baseline characteristics, no differences in survival were observed between lymphoma patients with and without synchronous solid tumors (*p* = 0.664).

**Conclusions:**

This is the first study to present the different situations of identifying SPMTs in lymphoma patients with synchronous solid tumors. In only <50% patients, SPMTs were identifiable at baseline. SPMT identification at different situations may make it difficult to choose the optimal therapeutic option, which may consequently impact patient survival.

## Graphical abstract

In the real‐world setting, there were three distinct situations in identifying second primary malignant tumors (SPMTs) in lymphoma patients with synchronous solid tumors. Only less than half of the patients confirmed twomalignancies at baseline. Others were unexpectedly identified SPMTs during surgery or chemotherapy.

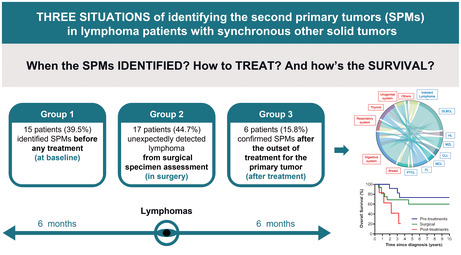

## 1 INTRODUCTION

The concept of multiple primary malignant tumors (MPMTs) was first proposed by Warren and Gates in 1932. MPMTs can be defined as the presence of ≥2 histologically distinct malignant tumors that are not due to recurrence, metastasis, or local spread in the same individual.^1^ Depending on the time interval of diagnosis between the first and second primary tumor, MPMTs can be divided into “synchronous” or “metachronous” categories. However, the exact time interval remains controversial. In current clinical practice, the time interval of 6 months is the most acknowledged, whereas a few studies define synchronous tumors with shorter time intervals.^2^, ^3^, ^4^

The synchronous presence of lymphoma and other tumors is rare. The incidence of synchronous tumors is approximately 0.8% in patients with non‐Hodgkin lymphoma.^5^ In case of these patients, decision‐making regarding optimal treatment modalities is challenging. In particular, for patients with an aggressive lymphoma course, the occurrence of other malignancies often increases the complexity of formulating an effective treatment plan. To date, “lymphoma first?” or “solid tumor first?” remains a dilemma. Treating malignant tumors with more aggressive biological behavior is usually preferred.^6^

However, in daily clinical practice, second primary malignant tumors (SPMTs) can be detected after antitumor treatment initiation. In some patients, SPMTs have been reported to be accidentally identified during postoperative pathologic examination^7^ or during treatment for the primary tumor,^8^, ^9^ which makes choosing the optimal treatment strategy more difficult. It is essential to recognize different situations of SPMT identification considering the implications on subsequent therapeutic strategies.^10^ To the best of our knowledge, only a few case reports or studies, most with small sample sizes, have explored the frequency and prognosis of synchronous SPMTs in patients with lymphoma^9^, ^11^, ^12^, ^13^; SPMT identification has rarely been investigated in this population of patients.^10^ Clinicians seek more knowledge pertaining to such rare cases. Therefore, we herein investigated the situations of identifying SPMTs at different treatment phases in lymphoma patients with synchronous solid tumors, and also explored patient characteristics, subsequent therapeutic strategies, and survival.

## 2 MATERIALS AND METHODS

### 2.1 Study population

This study was approved by the Ethics Committee of Peking University Cancer Hospital & Institute. We retrospectively reviewed the records of 5721 patients with lymphoma (International Classification of Diseases, 10th Revision, Clinical Modification [ICD‐10‐CM]: C81.0x–C85.9x) at Peking University Cancer Hospital & Institute, China, between February 2009 and May 2019. We included patients with synchronous solid tumors, which were diagnosed no more than 6 months before or after the diagnosis of lymphoma; this was reconfirmed by the experts at the Department of Pathology at Peking University Cancer Hospital & Institute. Herein we defined SPMTs as tumors that were identified later in this specific patient population. Detailed follow‐up data were obtained from outpatient records or by telephone interview, and causes of death were extracted from clinical records or obtained from patient family members. Based on the phases of SPMT recognition, we classified all enrolled patients into three groups: pretreatment group (group 1), which included patients in whom lymphoma and other solid tumors were concurrently identified before treatment; surgical group (group 2), which included patients in whom lymphoma was unexpectedly detected on surgical specimen assessment; and post‐treatment group (group 3), which included patients in whom SPMTs were detected after the outset of treatment for the primary tumor, excluding the situation of surgical confirmation.

Lymphoma was classified as advised by the World Health Organization^14^ and risk stratification was evaluated based on the International Prognostic Index.^14^, ^15^ Aggressive lymphomas referred to diffuse large B‐cell lymphoma (DLBCL), Hodgkin lymphoma, mantle cell lymphoma, follicular lymphoma (grade 3b), transformed indolent lymphoma, mediastinal large B‐cell lymphoma, and enteropathy‐associated T‐cell lymphoma. Furthermore, indolent lymphomas referred to follicular lymphoma (grade 1, 2, and 3a), marginal zone lymphoma, and chronic lymphocytic leukemia/small cell B‐cell lymphoma. The stage of lymphoma was determined using the Ann Arbor staging system.^16^, ^17^ Other solid tumors were restaged according to the 8th edition of the UICC/AJCC staging systems.^18^

### 2.2 Statistical analysis

Continuous variables were described as medians with interquartile ranges and compared using the Mann–Whitney *U*‐test. Categorical variables were assessed by Pearson chi‐square or Fisher's exact test. The survival endpoints of interest were overall survival (OS) and disease‐specific survival (DSS). OS was defined as the length of time from the initial diagnosis of tumor to death due to any cause, while DSS was defined as the length of time from diagnosis to death due to lymphoma/other solid tumors. DSS and OS were estimated using the Kaplan–Meier (KM) method. We also calculated “1 − KM” to estimate specific disease‐related cumulative hazards. Considering the occurrence of competing risk between tumors, we used the non‐parametric Aalen–Johansen estimator to calculate cumulative incidence function (CIF).^19^

Two‐sided *p* < 0.05 was considered statistically significant. Statistical analyses were performed using SPSS Statistics v25.0 (IBM Corp.) and R v3.2.5 (https://www.r‐project.org). The main R packages used were “survival,” “cmprsk,” and “MatchIt.”

To compare survival differences between lymphoma patients with or without synchronous tumors, we performed propensity score‐matched analysis with a database of 3739 lymphoma patients who previously represented the control group.^20^ One‐to‐three nearest neighbor matching without replacement was applied to balance the baseline characteristics of lymphoma patients (age at diagnosis, stage, and histological subtype), with a caliper of 0.1 of the standard deviation of the logit of the propensity score.^21^

## 3 RESULTS

### 3.1 Baseline clinical characteristics

We enrolled 38 patients (0.7%) with lymphoma and synchronous solid tumors (Supplementary Figure S1). The ratio of male to female was approximately 1:1, and the median age at the diagnosis of lymphoma was 62 years (range: 13–84 years; Supplementary Figure S2). Overall, 21.1% patients had a family history of cancer (Table 1).

**TABLE 1:** Baseline characteristics of patients

Characteristics	Group 1 no. (%) (*n* = 15)	Group 2 no. (%) (*n* = 17)	Group 3 No. (%) (*n* = 6)	*p* *	Total no. (%) (*n* = 38)
Age, years					
<45	0 (0.00)	1 (0.59)	1 (16.67)	0.255	2 (52.63)
≥45 & <60	7 (46.67)	6 (35.29)	1 (16.67)		14 (36.84)
≥60	8 (53.33)	10 (58.82)	4 (66.67)		22 (57.89)
Male	10 (66.67)	5 (29.41)	3 (50.00)	0.108	18 (47.37)
Tumor family history	2 (13.33)	4 (23.53)	2 (33.33)	0.564	8 (21.05)
Lymphomas^a^					
Aggressive lymphoma	9 (60.00)	10 (58.82)	3 (50.00)	0.911	22 (57.89)
Indolent lymphoma	6 (40.00)	7 (41.18)	3 (50.00)		16 (42.11)
Stage^b^					
I–II	4 (26.67)	5 (29.41)	2 (33.33)	0.941	11 (28.95)
III–IV	11 (73.33)	11 (64.71)	4 (66.67)		26 (68.42)
NC	0 (0.00)	1 (5.88)	0 (0.00)		1 (2.63)
LDH					
Normal	9 (60.00)	12 (70.59)	4 (66.67)	0.730	25 (65.79)
Elevated	5 (33.33)	4 (23.53)	1 (16.67)		10 (26.32)
NC	1 (6.67)	1 (5.88)	1 (16.67)		3 (7.89)
ECOG					
0	12 (80.00)	14 (82.35)	5 (83.33)	0.978	31 (81.58)
1–2	3 (20.00)	3 (17.65)	1 (16.67)		7 (18.40)
Extranodal sites					
≤1	13 (86.67)	13 (76.47)	6 (100.00)	0.210	32 (84.21)
>1	2 (13.33)	3 (17.65)	0 (0.00)		5 (13.16)
NC	0 (0.00)	1 (5.88)	0 (0.00)		1 (2.63)
IPI score					
0–1	6 (40.00)	7 (41.18)	3 (50.00)	0.552	16 (42.11)
2	4 (26.67)	4 (23.53)	2 (33.33)		10 (26.32)
3	4 (26.67)	2 (11.76)	1 (16.67)		7 (18.42)
4–5	0 (0.00)	3 (17.65)	0 (0.00)		3 (7.89)
NC	1 (6.67)	1 (5.88)	0 (0.00)		2 (5.26)
Other solid tumor stage^c^					
I–II	12 (80)	12 (70.59)	4 (66.67)	0.621	28 (73.68)
III–IV	2 (13.33)	3 (17.65)	2 (33.33)		7 (18.42)
NC	1 (6.67)	2 (11.76)	0 (0.00)		3 (7.89)

*Note*: Group 1: Patients in whom lymphoma and other solid tumors were concurrently identified before any antitumor treatments; Group 2: Patients in whom lymphoma was unexpectedly detected on surgical specimen assessment; Group 3: Patients in whom SPMTs were detected after the outset of treatment for the primary tumor, excluding the situation of surgical confirmation.

Abbreviations: ECOG, Eastern Cooperative Oncology Group; IPI, International Prognostic Index; LDH, lactate dehydrogenase; NC, not communicated.

^a^ Aggressive lymphomas referred to diffuse large B‐cell lymphoma, Hodgkin lymphoma, mantle cell lymphoma, follicular lymphoma (grade 3b), transformed indolent lymphoma, mediastinal large B‐cell lymphoma, and enteropathy‐associated T‐cell lymphoma. Indolent lymphomas referred to histologically confirmed grade 1, 2, and 3a follicular lymphoma, marginal zone lymphoma, and chronic lymphocytic leukemia/small cell B‐cell lymphoma.

^b^ Stage of lymphoma was evaluated using the Ann Arbor staging system.

^c^ Other solid tumors were restaged according to the 8^th^ edition of the UICC/AJCC staging systems.

* Missing data not included. *p* < 0.05 indicated statistically significant differences.

### 3.2 SPMT identification

**FIGURE 1:**
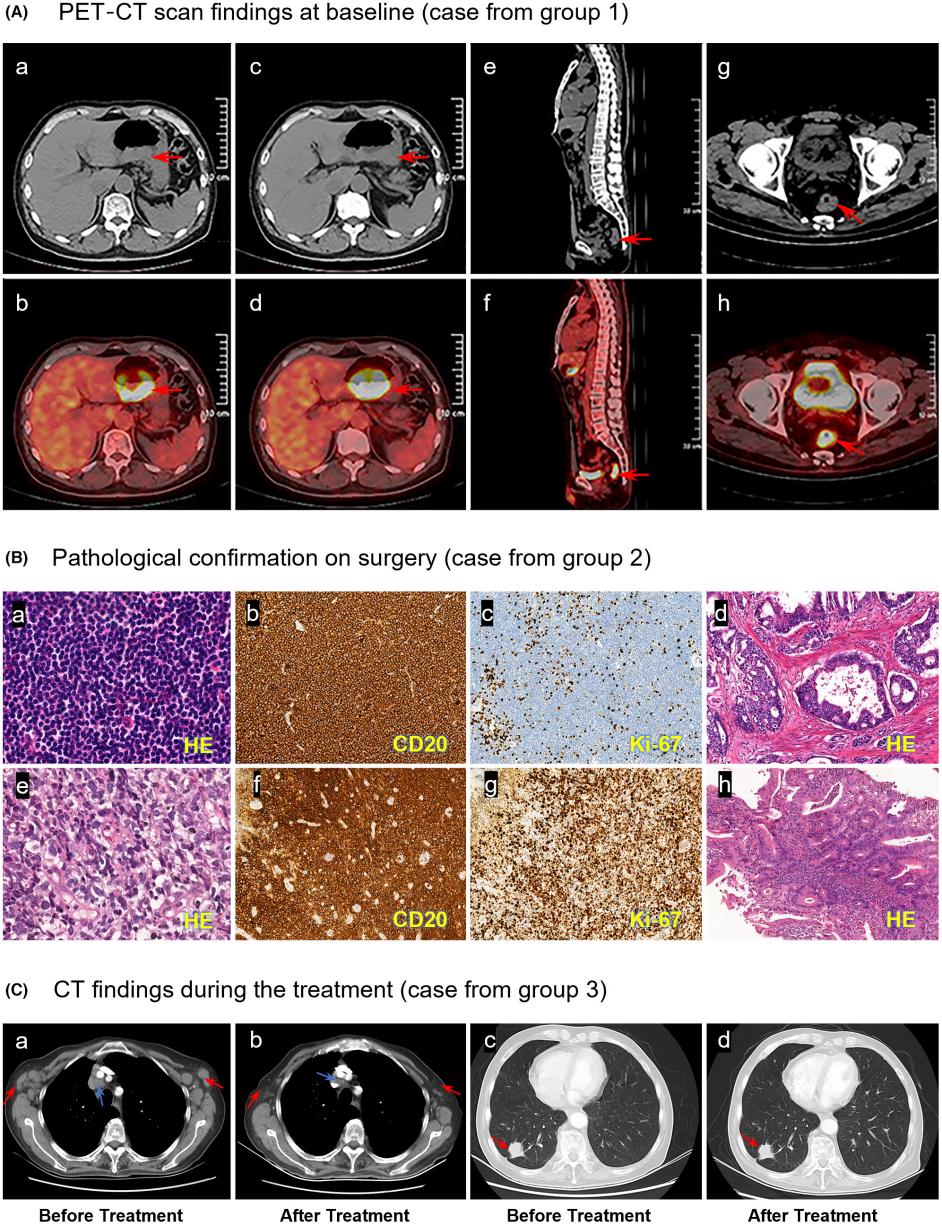
Case presentation of second tumor detection. A, PET/CT at baseline in a patient (no. 29) shows gastric wall thickening on the side of the gastric lesser curvature (arrows in a–d) and the rectum (arrows in e–h). Gastric diffuse large B‐cell lymphoma and colorectal adenocarcinoma were confirmed on subsequent gastrointestinal endoscopy before antitumor therapy initiation. B, a–d present marginal zone lymphoma with adenocarcinoma in a gastric specimen obtained from patient no. 11. Pathological sections reveal infiltration of diffuse small lymphocytes (hematoxylin–eosin [HE] staining, ×400; a), showing anti‐CD20 stain and Ki‐67 index of 20% (×100; b and c). Adenocarcinoma cells are arranged in glandular or cribriform structures (HE staining, ×100; d). e–h show a case (no. 18) of diffuse large B‐cell lymphoma not otherwise specified (HE, ×400; e), with positive expression of CD20 (×100; f) and high Ki‐67 index (×100; g), and well‐differentiated adenocarcinoma (×100; h) in the gastric mucosa. C, Contrast‐enhanced CT shows a significant reduction of mediastinal (blue arrows in a and c) and bilateral axillary (red arrows in a and c) lymph nodes after two cycles of the rituximab, cyclophosphamide, pegylated liposomal doxorubicin, vincristine, and prednisone regimen in a patient (no. 12) with DLBCL. However, the solid mass‐like lesion in the right lower lung lobe at the outer basal segment near the pleura appears similar to the previous one, with unchanged pleural traction signs (red arrows in b and d). Subsequent lung biopsy confirmed the diagnosis of lung adenocarcinoma. The patient continued treatment for lung cancer after finishing complete courses of the lymphoma chemotherapy regimen (6 cycles).

Lymphoma and other solid tumors were concurrently detected in 15 patients before any antitumor treatments were initiated (group 1; case presentation in Figure 1A). Besides, SPMTs were identified in 60.5% patients (*n* = 23) when antitumor treatments, including surgery, had been initiated. Among them, 17 patients underwent surgery for the primary solid malignant tumor, but synchronous lymphoma was then unexpectedly confirmed on surgical specimen assessment (group 2). Finally, six patients (15.8%) were diagnosed with SPMTs after the outset of treatment for the primary tumor (group 3; case presentation in Figure 1C).

### 3.3 Tumor subtypes

DLBCL was the most common histological type of lymphoma, followed by follicular lymphoma, marginal zone lymphoma, and mantle cell lymphoma. With regard to solid tumors, colorectal cancer and thyroid cancer were the most frequent, followed by gastric cancer and lung cancer (Table 2).

**TABLE 2:** Pathological subtypes of lymphomas and solid tumors

Solid tumor	Aggressive lymphoma no. (%)				Indolent lymphoma no. (%)			Total
	DLBCL	MCL	HL	ETL	FL	CLL/SLL	MZL	
Colorectal cancer	4 (10.53)	1 (2.63)	1 (2.63)	0 (0.00)	2 (5.26)	1 (2.63)	0 (0.00)	9 (23.68)
Gastric cancer	3 (7.89)	0 (0.00)	0 (0.00)	0 (0.00)	0 (0.00)	0 (0.00)	2 (5.26)	5 (13.16)
Esophageal cancer	1 (2.63)	0 (0.00)	0 (0.00)	0 (0.00)	0 (0.00)	0 (0.00)	0 (0.00)	1 (2.63)
Lung cancer	2 (5.26)	1 (2.63)	0 (0.00)	0 (0.00)	0 (0.00)	1 (2.63)	0 (0.00)	4 (10.53)
Bladder cancer	0 (0.00)	0 (0.00)	0 (0.00)	0 (0.00)	1 (2.63)	1 (2.63)	0 (0.00)	1 (2.63)
Breast cancer	3 (7.89)	0 (0.00)	0 (0.00)	0 (0.00)	0 (0.00)	0 (0.00)	0 (0.00)	4 (10.53)
Kidney cancer	1 (2.63)	0 (0.00)	0 (0.00)	1 (2.63)	0 (0.00)	0 (0.00)	0 (0.00)	2 (5.26)
Thyroid cancer	2 (5.26)	2 (5.26)	1 (2.63)	0 (0.00)	3 (7.89)	1 (2.63)	0 (0.00)	9 (23.68)
Eyelid cancer	0 (0.00)	0 (0.00)	0 (0.00)	0 (0.00)	1 (2.63)	0 (0.00)	0 (0.00)	1 (2.63)
Ovarian cancer	0 (0.00)	0 (0.00)	0 (0.00)	0 (0.00)	0 (0.00)	0 (0.00)	1 (2.63)	1 (2.63)
Cholangiocellular Cancer	0 (0.00)	0 (0.00)	0 (0.00)	0 (0.00)	0 (0.00)	0 (0.00)	1 (2.63)	1 (2.63)
Total	16 (42.11)	4 (10.53)	2 (5.26)	1 (2.63)	7 (18.42)	4 (10.53)	4 (10.53)	38 (100.00)

Abbreviations: CLL/SLL, chronic lymphocytic leukemia/small cell B‐cell lymphoma; DLBCL, diffuse large B‐cell lymphoma; EATL, Enteropathy‐associated T‐cell lymphoma; FL, Follicular lymphoma (grade 1, 2, and 3a); HL, Hodgkin lymphoma; MCL, mantle cell lymphoma; MZL, marginal zone lymphoma.

**FIGURE 2:**
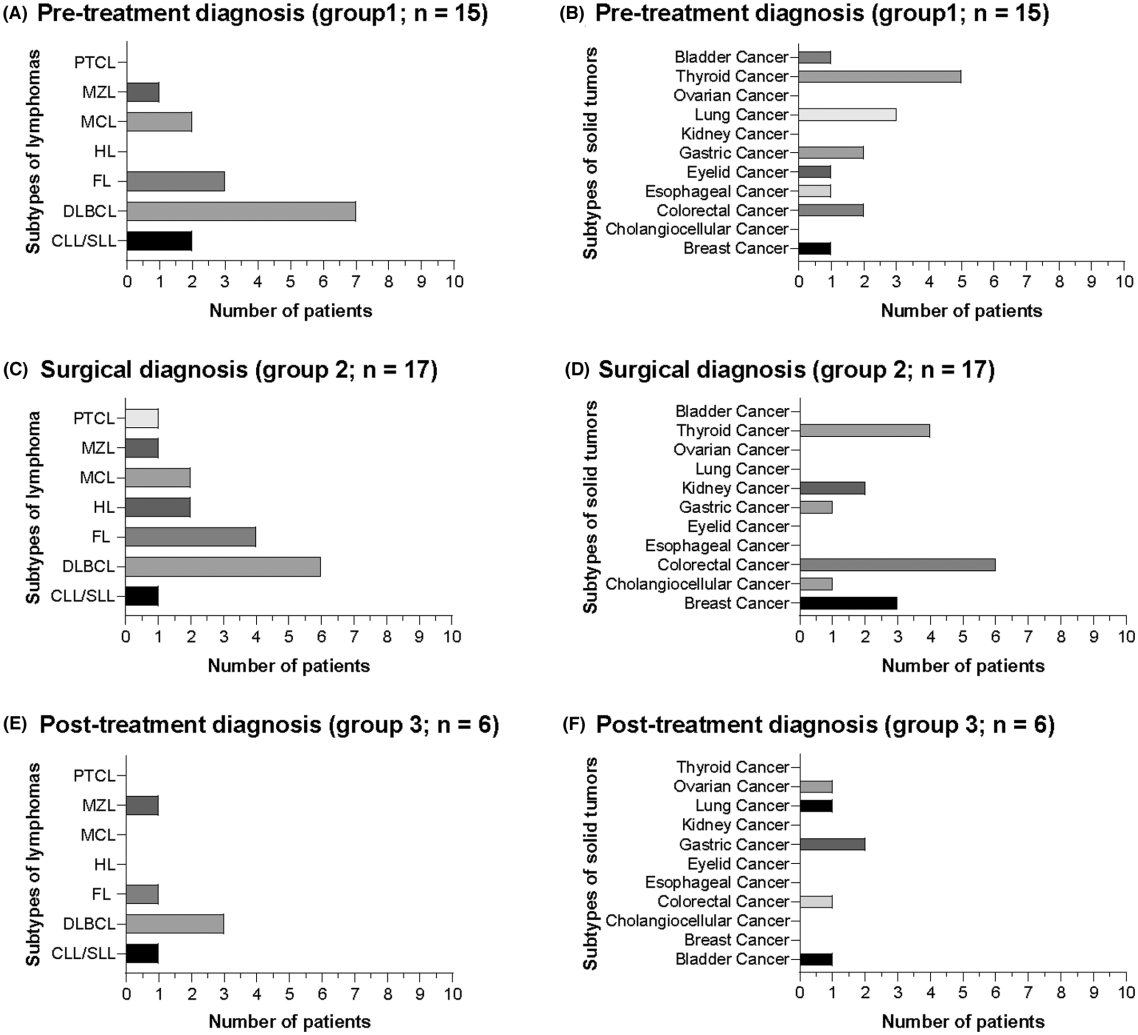
Distribution of tumor subtypes in different groups. We divided 38 patients with lymphoma and synchronous tumors into three groups, depending on when the presence of SPMTs was confirmed: pretreatment (group 1), surgical (group 2), and post‐treatment (group 3) groups. Subtypes of lymphoma and solid tumors are presented for group 1 (*n* = 15, A and B), 2 (*n* = 17, C and D), and 3 (*n* = 6, E and F)

The most common solid tumor in groups 1, 2, and 3 was thyroid cancer, colorectal cancer, and gastric cancer, respectively. DLBCL was the most common lymphoma subtype in all three groups (Figure 2). Interestingly, two tumor components of different morphology could be observed in the same surgical specimen for five patients in group 2 (Figure 1B[e–h]).

### 3.4 Treatment options

**FIGURE 3:**
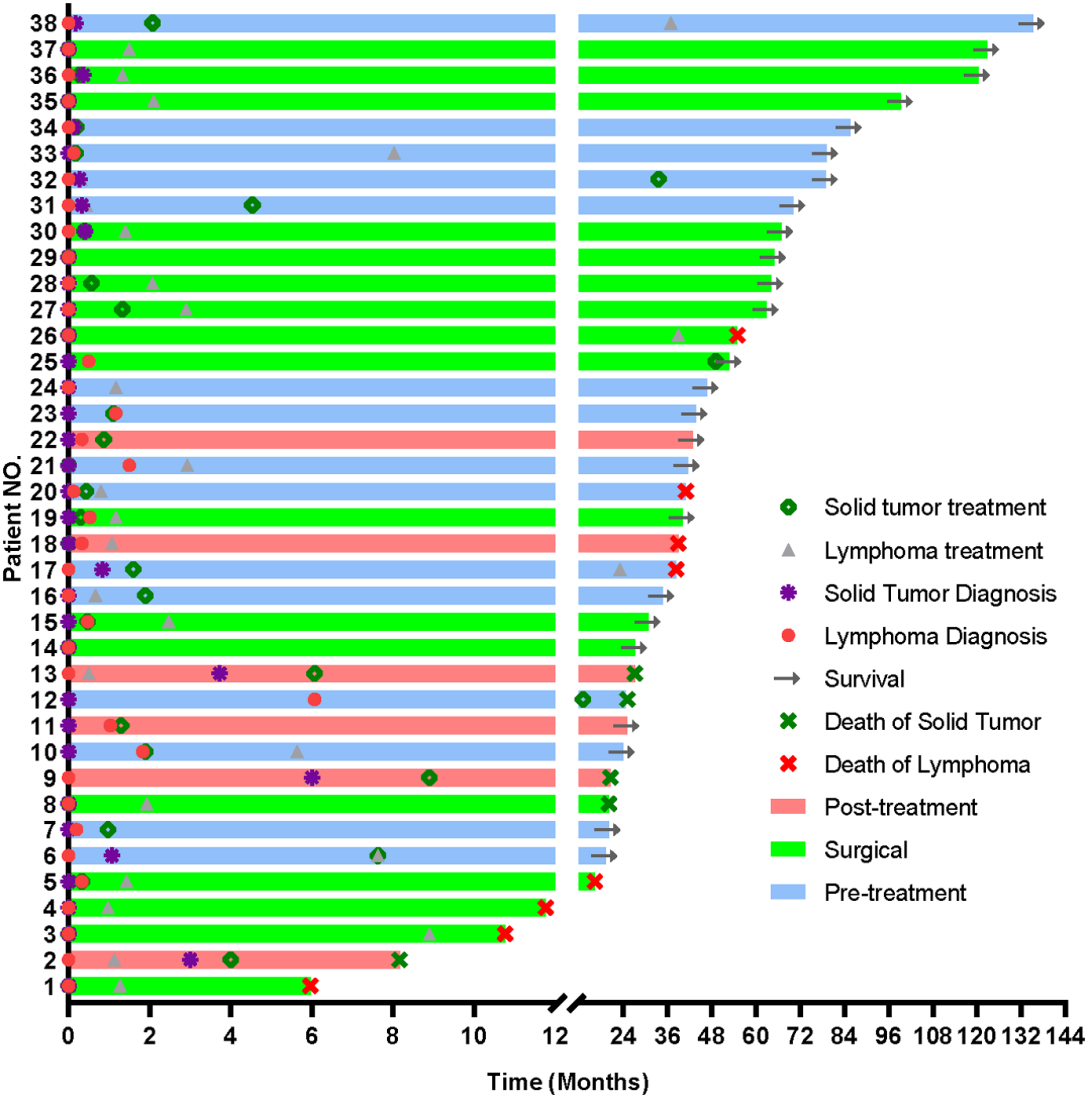
Diagnosis, treatments, and outcomes for 38 patients with lymphoma and synchronous solid tumors

Subsequent treatment choices widely varied (Figure 3). In group 1 (*n* = 15), among nine patients with aggressive lymphoma, six started initial treatments for other solid tumors first, whereas in case of three patients, priority was given to anti‐lymphoma treatment.

In group 2, for patients in whom lymphoma was unexpectedly identified (*n* = 17), most patients with aggressive lymphoma (9/10) and > 50% patients with indolent lymphoma (4/7) received anti‐lymphoma chemotherapy after surgery.

In group 3, in four patients with lymphoma, other solid tumors were identified after the initiation of chemotherapy. Of them, three switched toward the treatment plan for SPMTs (two patients with gastric cancer and one with ovarian cancer), whereas the remaining one patient continued anti‐lymphoma therapy, despite the detection of early‐stage lung cancer. In two patients with other solid tumors (one with bladder cancer and the other with colorectal cancer), anti‐lymphoma therapy was not initiated, despite the identification of indolent lymphoma.

### 3.5 Survival analysis

**FIGURE 4:**
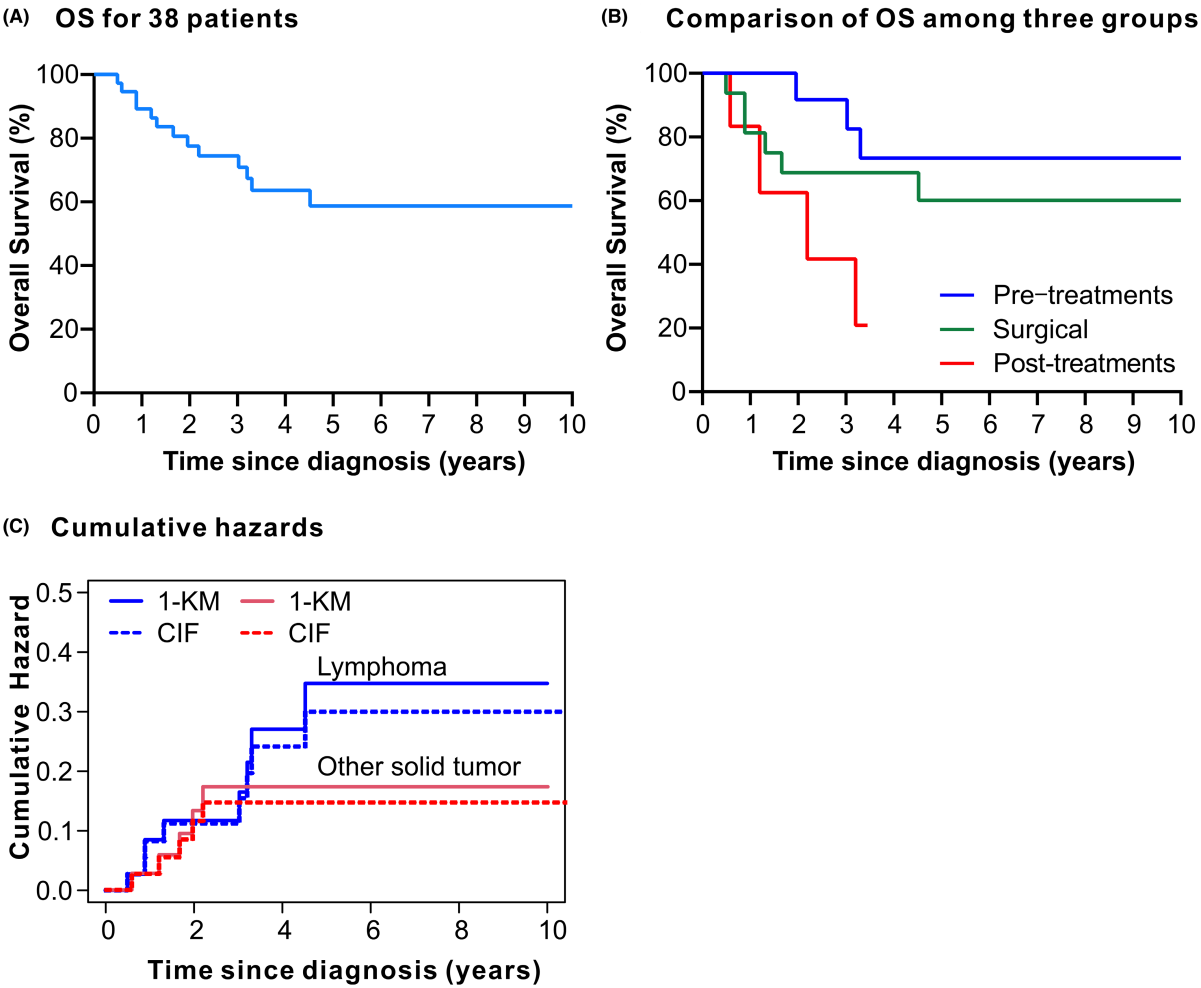
Overall survival and cumulative hazards. OS, overall survival; CIF, cumulative incidence function; KM, Kaplan–Meier. A, OS curve for all 38 patients with lymphomas and synchronous other solid tumors. B, there was a statistical difference among the three groups (*p* = 0.048, *p* for trend = 0.025). The pretreatment group appeared to have better prognosis than the other two groups. C, the estimates calculated using the KM method might have overestimated the actual risk, and differences in cumulative hazard estimates between the CIF and KM methods became more apparent with time.

The median follow‐up was 5.0 years and 13 (34.2%) patients died (Supplementary Table S1), and the median OS was not reached. The expected 5‐year OS was 58.7% [95% confidence interval (CI), 43.2%–79.8%; Figure 4A] for the entire cohort, 73.3% (95% CI: 51.5%–NA) for group 1, 60.2% (95% CI: 39.5%–91.7%) for group 2, and 20.8% (95% CI: 3.68%–NA) for group 3. OS showed significant differences among the three groups (log‐rank = 6.053, *p* = 0.048, *p* for trend = 0.025; Figure 4B).

On considering the competing risk between lymphoma and synchronous solid tumors, CIF within 5 years was 26.6% for lymphoma and 14.7% for other solid tumors (Figure 4C). However, specific disease‐related cumulative hazard at 5 years was 30.0% and 16.2% for lymphoma and other solid tumors, respectively, as evaluated using conventional methods (KM). As evident, these values were higher than CIF estimates (Supplementary Table S2).

The baseline characteristics of patients were balanced using the propensity score‐matched model between lymphoma patients with synchronous other solid tumors (n = 38) and control patients in the database (*n* = 114). However, no difference in survival was observed between the groups (log‐rank = 0.213, *p* = 0.664, Supplementary Table S3).

## 4 DISCUSSION

Although very rare, the prevalence of MPMTs is gradually increasing, which is attributable to the application of multiple approaches for tumor detection and the aging population.^22^, ^23^, ^24^, ^25^ We herein found that <50% SPMTs were identifiable at baseline in patients with synchronous lymphoma and other solid tumors, revealing the challenges associated with their early recognition in the clinical setting.

Advancements in diagnostic technologies have improved the identification of MPMTs. Multidetector CT,^26^ PET/CT,^27^ PET/MRI,^28^ and endoscopy^29^ have been reported to be highly efficient at detecting synchronous tumors. Molecular profiling is also a useful diagnostic tool, particularly when differential diagnosis between SPMTs and metastasis cannot be confirmed by histomorphology.^30^, ^31^, ^32^ We found that nine patients had a family cancer history and three showed a third distinct malignant tumor in this cohort. Wang et al. recently reported germline variants of DNA repair genes and of genes involved in B cell functions to be potential causes of MPMTs.^33^ Further studies on this topic are warranted.

Patients in whom SPMTs were identified before treatment initiation (group 1) are highly likely to benefit from multidisciplinary team discussions and individualized therapy plans.^34^ Furthermore, in group 1, early‐stage papillary thyroid cancer was the dominant histological subtype of solid tumors, while in groups 2 and 3, gastrointestinal adenocarcinoma was the most prevalent. The distinct biological behavior of solid tumors influences patient outcomes.^35^, ^36^ These reasons may partly explain the relatively good prognosis in group 1.

Data pertaining to group 2 indicated that lymphoma was most likely to be accidentally identified when patients underwent surgery for colorectal cancer or thyroid. Similar cases have been previously reported.^12^, ^37^ Almost all patients with aggressive lymphoma (9/10) in this group received anti‐lymphoma chemotherapy after surgery, and > 50% (6/10) underwent radical surgery for early‐stage solid tumors. Thus, they are more likely to receive anti‐lymphoma chemotherapy after therapy.

In case of six patients, SPMTs were detected during/after the outset of treatment for the primary tumor (group 3). Gastric cancer was the most common second solid tumor during the treatment for lymphoma, suggesting that endoscopy is essential for lymphoma patients with gastrointestinal lesions. In case of two patients with lymphoma, the treatment scheme was adjusted for gastriccancers, whereas in one patient with DLBCL, anti‐lymphoma treatment was continued after lung adenocarcinoma was confirmed at an early stage. Although difficult, it is crucial to distinguish the SPMTs in patients with poor response from distant metastasis of the first malignancy.^10^ Considering that therapeutic options for the initial treatment might be limited, such patients may show relatively worse prognosis. In addition, the prevalence of early‐stage papillary thyroid cancer was approximately 40% and 30% in group 1 and 2, respectively, and 0% in group 3. However, 50% patients were diagnosed with gastrointestinal lesions in group 3. The distribution of solid tumors may be another possible reason for the poor outcome in group 3 (Figure 2).

There is still a lack of standard treatment guidelines for lymphoma patients with synchronous other malignant tumors. But the therapeutic patterns and pharmacological agents are partly overlapped in clinical practice. Platinum‐based chemotherapy is reportedly effective for treating multiple tumors.^38^, ^39^ One patient with DLBCL and lung adenocarcinoma received pemetrexed and carboplatin as the first line of treatment, while another patient with DLBCL and synchronous gastric cancer received gemcitabine plus oxaliplatin, similar to a previously reported case.^9^ In addition, anthracyclines are commonly used to treat multiple tumors, but dose‐limiting toxicity needs to be considered when using them to treat MPMTs.^40^, ^41^ To summarize, therapy choices widely vary in most cases, despite tumors showing the same characteristics in the real‐world setting.

Herein we also performed competing analyses to evaluate the cumulative incidence of lymphoma and solid tumors. SPMTs have been analyzed as competing risks in patients with, for example, esophageal cancer,^42^ thyroid cancer,^43^ and head and neck squamous cell carcinoma,^44^ but not in those with synchronous lymphoma and other malignancies. The estimates calculated using the KM method might overestimate actual specific disease‐related cumulative hazards, which has been previously reported,^45^, ^46^ supporting that the non‐parametric Aalen–Johansen estimator may generate a more unbiased estimate of CIF (Supplementary Table S1). In addition, we did not find a statistical survival difference between lymphoma patients with and without synchronous solid tumors after matching, which could be due to the small sample size. However, in comparison with 5‐year OS of 62% for all lymphomas at our center,^20^, ^47^ lymphoma patients with synchronous/metachronous tumors may show worse long‐term outcomes.

This study has some limitations. First, considering the small sample size, it is challenging to draw robust conclusions. All pertinent results reported in this study are preliminary and exploratory. It is notable that considering the rarity of synchronous lymphoma and other solid tumors, to the best of our knowledge, the sample size assessed in this study is currently the largest. Second, patients were enrolled in this retrospective study for approximately 10 years, along with potential time‐varying confounders.^48^ In recent years, the methods to treat malignant tumors have markedly changed with the advent of novel strategies, such as immunotherapies,^49^ targeted therapies,^50^, ^51^ and epigenetic therapies.^52^ Therefore, we did not analyze any subsequent specific treatment patterns. Further studies with a larger sample size are accordingly warranted to substantiate our findings.

## 5 CONCLUSIONS

There were three distinct situations of identification of SPMTs in lymphoma patients with synchronous other solid tumors in the real‐world setting. SPMTs could be unexpectedly identified during surgery or chemotherapy, whereas only <50% were identifiable at baseline. The early diagnosis of SPMTs remains challenging in clinical practice. The application of multiple diagnostic techniques can perhaps facilitate their early detection; furthermore, the multi‐modalities for treating this population are complex but continue to evolve.

## AUTHOR CONTRIBUTIONS

Weiping Liu and Jun Zhu conceptualized this research. Hongye Gao and Xinqiang Ji collected data. Hongye Gao performed the analysis and prepared the original draft, figures, and tables. Yumei Lai conducted the histological examination of synchronous tumors and interpreted pathological results. Xiaogan Wang provided helpful suggestions. All authors have reviewed, revised, and approved the final manuscript.

## CONFLICT OF INTEREST

The authors declare that they have no competing interests.

## ETHICAL APPROVAL STATEMENT

This retrospective study was approved by the Ethics Committee of Peking University Cancer Hospital & Institute, and waiver of informed consent was granted due to anonymized use of data (all data were handled with strict anonymity). All study procedures were performed in line with the Declaration of Helsinki. This study did not involve any experiments with animals.

## CLINICAL TRIAL REGISTRATION NUMBER

Not applicable.

## Supporting information


Data S1

## Data Availability

Dataset will be made available from the corresponding author on reasonable request.
