# Supplementary material for: Different situations of identifying second primary malignant tumors in lymphoma patients with synchronous solid tumors
Source: Cancer Med. 2023 Jan 9;12(7):8038–49. doi: 10.1002/cam4.5592 (PMC10134266; doi:10.1002/cam4.5592)
Supplement: Supplementary file 1 — Data S1 [file CAM4-12-8038-s001.docx]

Supplementary Figure 1 Study flow diagram

**
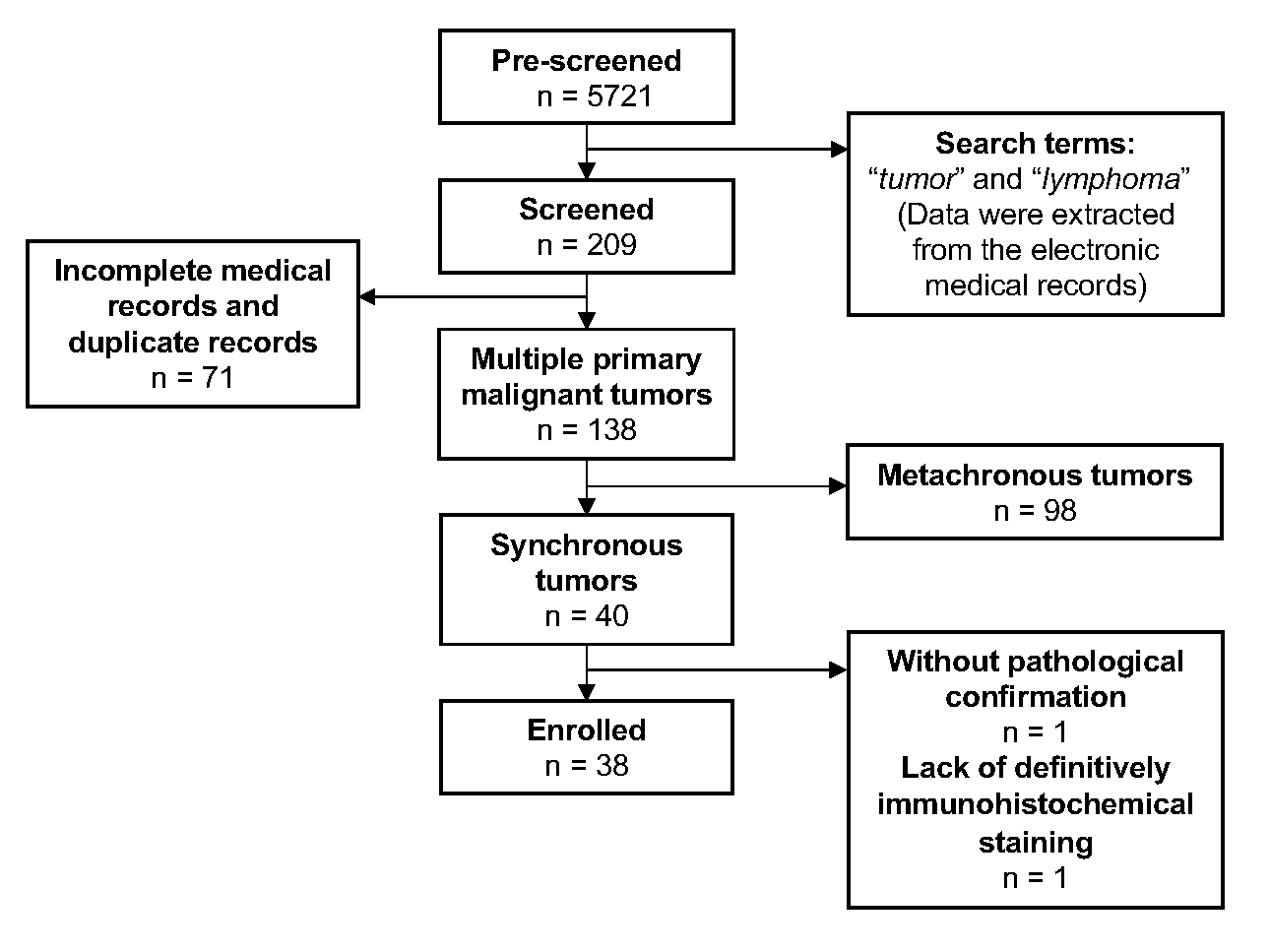
**

Supplementary Figure 2 Distribution of age and number of patients who diagnosed with lymphoma and synchronous tumors

**
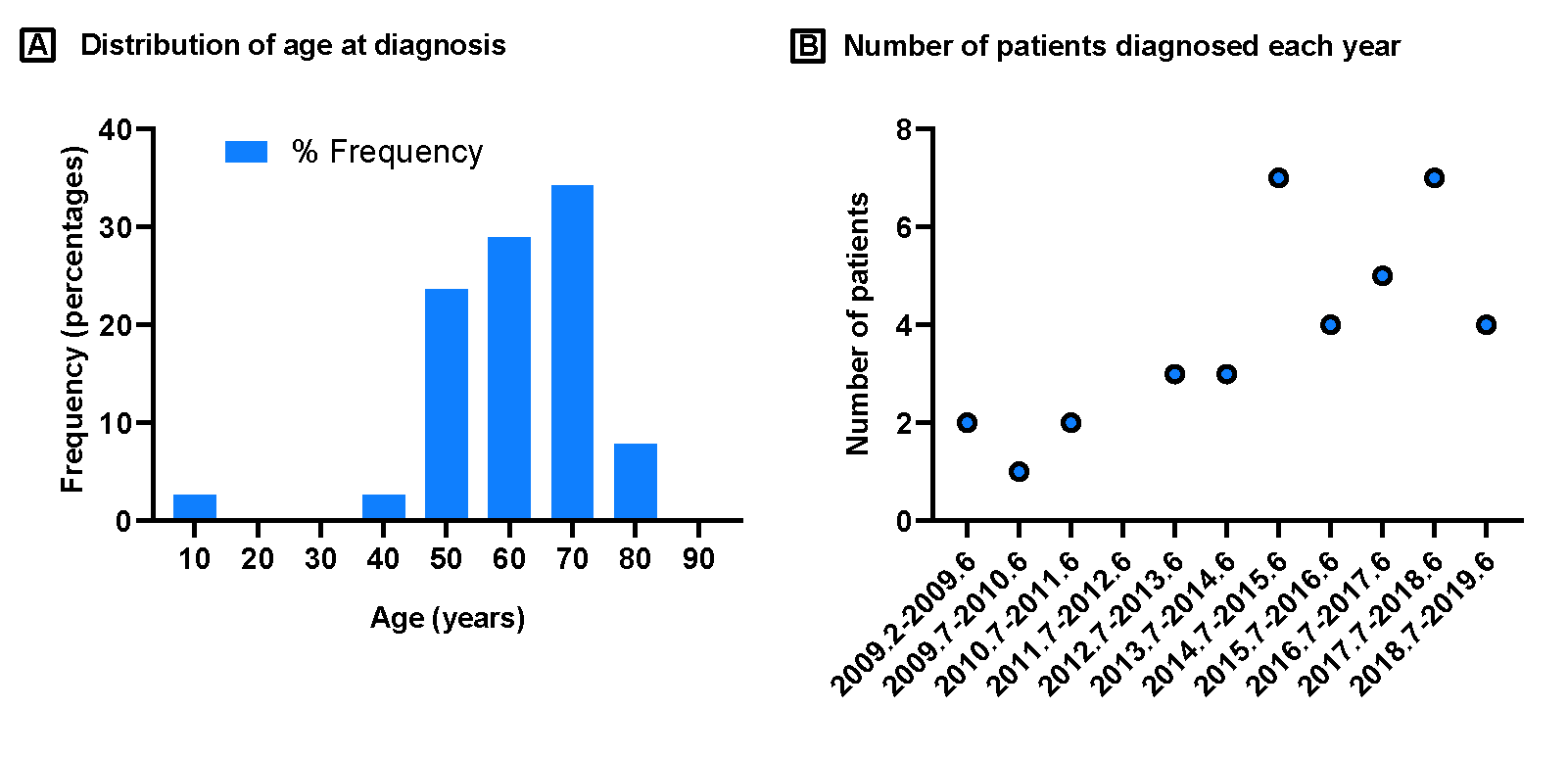
**

Supplementary Table 1 Cumulative hazards calculated by two methods

|  | **Cumulative Hazards** | | | | |
| --- | --- | --- | --- | --- | --- |
|  | 1-year | 2-year | 3-year | 4-year | 5-year |
| **Lymphoma** |  |  |  |  |  |
| CIF | 0.081 | 0.109 | 0.109 | 0.217 | 0.266 |
| 1- KM | 0.083 | 0.112 | 0.154 | 0.241 | 0.300 |
| **Other solid tumor** |  |  |  |  |  |
| CIF | 0.027 | 0.116 | 0.147 | 0.147 | 0.147 |
| 1- KM | 0.028 | 0.127 | 0.162 | 0.162 | 0.162 |

**CIF**: cumulative incidence function; **KM**: Kaplan‑Meier method

Supplementary Table 2 Cause of death

| **Cause of Death** | **Group 1**  **(n = 15)** | **Group 2**  **(n = 17)** | **Group 3**  **(n = 6)** | **Total**  **(n = 38)** |
| --- | --- | --- | --- | --- |
| Lymphoma | 2 (13.33%) | 5 (29.41%) | 1 (16.67%) | 8 (21.05%) |
| Other solid tumor | 1 (6.67%) | 1 (5.88%) | 3 (50.00%) | 5 (13.20%) |

**Group 1**: Patients who were identified lymphoma and other solid tumors concurrently before any anti-tumor treatments; **Group 2**: Patients who found the synchronous SPMs in the surgery for the primary solid malignant tumor but then confirmed synchronous lymphoma unexpectedly by surgical specimens; **Group 3**: Patients who were diagnosed with the SPMs after the outset of the treatment for the first primary tumor.

Supplementary Table 3 Baseline Characteristics before and after propensity score-matched model

| **Variable** | **Unmatched Data** | | |  | **Matched Data** | | |
| --- | --- | --- | --- | --- | --- | --- | --- |
|  | **Synchronous group**  **( n = 38)** | **Control group**  **(n = 3739)** | ***P* Value*** |  | **Synchronous group**  **( n = 38)** | **Control group**  **(n = 114)** | ***P* Value** |
| **Age, y** | 62.00 (51.5-70.25) | 50.25 (34.58-62.50) | < 0.001 |  | 62.00 (51.50-70.25) | 62.00 (51.50-70.25) | 0.964 |
| **Male** | 18 (47.4%) | 2177 (58.2%) | 0.177 |  | 18 (47.4%) | 54 (47.0%) | 1 |
| **Lymphomas** ^§^ |  |  | 0.001 |  |  |  | 0.293 |
| **Stage** † |  |  | 0.060 |  |  |  | 0.067 |
| I | 7 (18.4%) | 416 (11.1%) |  |  | 7 (18.4%) | 10 (8.8%) |  |
| II | 4 (10.5%) | 1012 (27.1%) |  |  | 4 (10.5%) | 31 (27.2%) |  |
| III | 6 (15.8%) | 732 (19.6%) |  |  | 6 (15.8%) | 24 (21.1%) |  |
| IV | 21 (55.3%) | 1579 (42.2%) |  |  | 21 (55.3%) | 49 (43.0%) |  |

Data are described as median (interquartile ranges) or numbers (%). Missing data did not include.

†The stage of lymphoma was graded based on the Ann-Arbor staging system.

* Age was compared with the Mann-Whitney *U* test for nonnormal distribution.

 Male, lymphoma (subtypes according to WHO, 2016), and stage were assessed by the Pearson chi-square or Fisher’s exact test. *P* < 0.05 was considered statistically different.

§We coded histologic subtypes of lymphoma and matched 2 groups with the same code.
